# DNA-PK inhibitor peposertib enhances p53-dependent cytotoxicity of DNA double-strand break inducing therapy in acute leukemia

**DOI:** 10.1038/s41598-021-90500-3

**Published:** 2021-06-09

**Authors:** Eric Haines, Yuki Nishida, Michael I. Carr, Rafael Heinz Montoya, Lauren B. Ostermann, Weiguo Zhang, Frank T. Zenke, Andree Blaukat, Michael Andreeff, Lyubomir T. Vassilev

**Affiliations:** 1 Translational Innovation Platform Oncology and Immuno-Oncology, EMD Serono Research & Development Institute, Inc, Billerica, MA USA; 2 Section of Molecular Hematology and Therapy, Department of Leukemia, The University of Texas MD Anderson Cancer Center, Houston, Texas USA; 3 Translational Innovation Platform Oncology and Immuno-Oncology, Merck KGaA, Darmstadt, Germany

**Keywords:** Cancer, Drug discovery, Molecular medicine, Oncology

## Abstract

Peposertib (M3814) is a potent and selective DNA-PK inhibitor in early clinical development. It effectively blocks non-homologous end-joining repair of DNA double-strand breaks (DSB) and strongly potentiates the antitumor effect of ionizing radiation (IR) and topoisomerase II inhibitors. By suppressing DNA-PK catalytic activity in the presence of DNA DSB, M3814 potentiates ATM/p53 signaling leading to enhanced p53-dependent antitumor activity in tumor cells. Here, we investigated the therapeutic potential of M3814 in combination with DSB-inducing agents in leukemia cells and a patient-derived tumor. We show that in the presence of IR or topoisomerase II inhibitors, M3814 boosts the ATM/p53 response in acute leukemia cells leading to the elevation of p53 protein levels as well as its transcriptional activity. M3814 synergistically sensitized p53 wild-type, but not p53-deficient, AML cells to killing by DSB-inducing agents via p53-dependent apoptosis involving both intrinsic and extrinsic effector pathways. The antileukemic effect was further potentiated by enhancing daunorubicin-induced myeloid cell differentiation. Further, combined with the fixed-ratio liposomal formulation of daunorubicin and cytarabine, CPX-351, M3814 enhanced the efficacy against leukemia cells in vitro and in vivo without increasing hematopoietic toxicity, suggesting that DNA-PK inhibition could offer a novel clinical strategy for harnessing the anticancer potential of p53 in AML therapy.

## Introduction

Over the past few decades, substantial research efforts have been focused on improving the disease outcomes in acute myeloid leukemia (AML) patients. However, the overall therapeutic success remains relatively poor, with a five-year survival of approximately 30%^1^. While venetoclax-based combinatorial therapies have allowed effective induction of remissions in elderly patients with AML^2–4^, chemotherapy remains a primary standard-of-care (SoC) option for many patients. However, its effectiveness is appreciably limited by the development of chemoresistance^5–7^. Disabling of the apoptotic response in leukemia cells has been considered as a key pathway for resistance, and therapies that can effectively restore or potentiate it are actively being developed^7–11^.

The DNA-dependent protein kinase (DNA-PK) is a key driver of one of the main pathways for repair of DNA double-strand breaks (DSB) via the non-homologous end-joining mechanism^12–14^. Inhibitors of its catalytic activity have been shown to potentiate the antitumor activity of DNA DSB-inducing agents such as ionizing radiation (IR) and anthracyclines, both widely used in the treatment of cancer^15–17^. The master tumor suppressor p53 plays an essential role in the cellular response to DNA damage, including DSB, by inducing cell cycle arrest and/or apoptosis^18–20^. In response to DSB, p53 is phosphorylated and activated by ATM kinase, which senses the DNA damage and activates checkpoint signaling to protect cells from its detrimental effects^21^.

Peposertib (M3814), is a novel, potent and selective DNA-PK inhibitor with optimized pharmacological properties^22,23^, which has completed phase 1 clinical evaluation^24^ and is currently undergoing investigation in solid tumors in combination with radiation and immunotherapy. We recently reported that p53 plays a critical role in determining the fate of irradiated solid tumor cells in the presence of M3814^23^. In cells expressing wild-type p53, DNA-PK inhibition by M3814 strongly enhanced radiation-induced ATM/p53 signaling, leading to a complete blockade of proliferation and premature senescence. The M3814-modified checkpoint response effectively protected cancer cells from the catastrophic consequences of cell cycle progression with unrepaired DSB. Cancer cells with unrepaired DNA DSBs and devoid of p53’s activity lose checkpoint protection, enter mitosis with severe chromosomal aberrations and die from mitotic catastrophe^23^. Despite enhanced p53 activation, most epithelial cancer cell lines expressing wild-type p53 are unable to undergo p53-dependent apoptosis when exposed to IR and M3814^23^. This is likely because p53’s apoptotic function is frequently lost in these cells^25^. However, unlike most solid tumors, p53 is rarely mutated in leukemia and its apoptotic potential is retained^9,10^. Therefore, the p53 activity enhancement by the combination of DNA-PK inhibition and DSB-inducing agents may offer a new approach to improving the therapeutic response in acute leukemias. We recently demonstrated that such mechanism is involved in the potentiation effect of M3814 on calicheamicin, the DSB-inducing, antitumor antibiotic of Mylotarg in AML cells^26^.

Here, we show that in the presence of DSB-inducing therapies, IR and topoisomerase II inhibitors, M3814 potentiates the ATM/p53 signaling in acute leukemia cells leading to elevation of p53 protein levels and transcriptional activity. M3814 effectively potentiates the antileukemic effect of topoisomerase II inhibitors by enhancing p53-dependent AML cell apoptosis. We show that M3814 can enhance the SoC AML treatment, daunorubicin (DNR) plus cytarabine (AraC), or their liposomal formulation, CPX-351, in p53 wild-type leukemia cells in vitro and in an in vivo PDX model of AML. The treatment was tolerated well by normal bone marrow stem cells cultivated in vitro and by the hematopoietic system of the experimental animals used in vivo. Our data support the hypothesis that intervention in DSB repair by selective DNA-PK inhibition may offer a novel, potentially more efficacious, combination strategy for harnessing the apoptotic potential of p53 in leukemia therapy.

## Results

### M3814 enhances ATM/p53 signaling in response to radiation in acute leukemia cells

M3814 intervenes in the response of cancer cells to IR by potentiation of the ATM pathway and its downstream signaling, including p53 levels and transcriptional activity. This boost in p53 activity has major consequences for cell survival and p53 plays a key role in determining the fate of irradiated cancer cells^23^. p53 activation induces cell cycle arrest in most epithelial tumor cell lines while it rarely triggers apoptosis, which is likely due to altered p53 apoptotic signaling^25^. However, p53 activation in acute leukemia cells by both genotoxic and non-genotoxic approaches has been shown to effectively kill these cells via p53-dependent apoptosis^9,10^.

**Figure 1:**
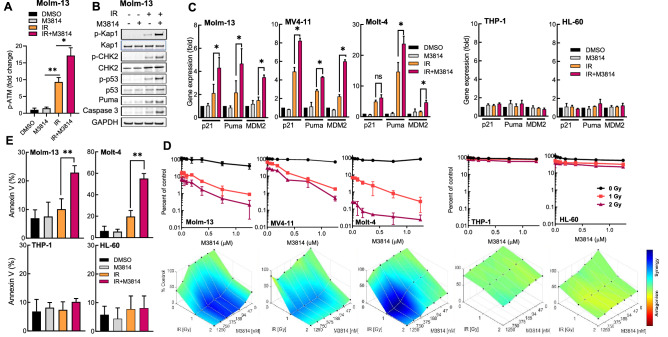
M3814 over-activates IR-induced p53 apoptotic signaling in acute leukemia cells. (**A**) Molm-13 cells were pre-treated with M3814 (1 µM) or vehicle for 45 min before exposure to IR (2 Gy). Protein lysates were isolated 4 h after irradiation and analyzed by MSD for phospho-ATM (Ser^1981^) and ATM. Results shown as fold change in p-ATM normalized to total ATM protein. (**B**) Molm-13 cells were treated as in A. Protein lysates were isolated 4 h after irradiation and analyzed by Western blotting using antibodies for phospho-Kap1(Ser^824^), Kap1, phospho-CHK2(Thr^68^), CHK2, phospho-p53(Ser^15^), p53, Puma, cleaved caspase-3 and GAPDH. Panel displays cropped images from repeat blots divided and probed for different targets. Full-sized blots are presented in Supplementary Fig. 1. (**C**) Molm-13, Molt-4, MV4-11, THP-1 and HL-60 cells were M3814-treated and irradiated as in A. RNA was isolated 4 h post-IR and p53 target gene expression was assessed by qPCR using probes for p21, MDM2 and Puma. Expression levels were normalized to GAPDH. (**D**) Molm-13, Molt-4, MV4-11, THP-1 and HL-60 cells were pre-treated with increasing M3814 concentrations for 45 min before exposure to IR (1 Gy or 2 Gy). Cell growth/viability was assessed via CellTiter Glo assay on d 4. Shown are individual M3814 dose–response curves (top) and overlays of Bliss synergy on relative viability landscapes (bottom). Bliss synergy was determined using Combenefit software. (**E**) Molm-13, Molt-4, THP-1 and HL-60 cells were treated as in A, then exposed to 1 Gy IR and 24 h later cells undergoing apoptosis were assessed by flow cytometry using a PE-conjugated annexin V. *p < 0.05, **p < 0.01, ***p < 0.001. NS: p > 0.05.

We examined the effect of the DNA-PK inhibitor on the p53 response to DSBs induced by IR in a small panel of acute leukemia cell lines, three of which express wild-type p53 (MOLM-13, Molt-4, MV4-11) and two are p53-deficient (THP-1 and HL-60). Proliferating Molm-13 cells were exposed to IR (2 Gy) and M3814 at a concentration shown to inhibit DNA-PK catalytic activity over 80% in multiple cancer cell lines (1 µM)^22,23^. As previously documented in epithelial cancer cell lines^23^, we observed higher levels of ATM activation as revealed by elevated phospho-ATM (Ser^1981^) in cells exposed to IR and M3814 compared to radiation alone (Fig. 1A). Enhanced ATM activity manifested as upregulation of its direct phosphorylation targets p-Kap1 (Ser^824^), p-CHK2 (Thr^68^) and p-p53 (Ser^15^) (Fig. 1B). Protein levels of both the key mediator of p53-dependent apoptosis, Puma, and cleaved caspase-3 were also elevated compared to radiation-only controls. M3814 alone did not show a marked effect on the levels of the examined proteins compared to the vehicle controls. In addition, Western blot analyses revealed increased apoptosis at a relatively early time-point (4 h). M3814-enhanced p53 activation led to a significantly increased expression of its transcriptional targets, p21, MDM2 and Puma, compared to IR alone only in the p53 wild-type (Molm-13, MV4-11, Molt-4) but not p53-deficient cell lines (THP-1, HL-60) (Fig. 1C). No statistically significant changes in gene expression were triggered by M3814 alone in all tested cell lines (Fig. 1C). These results indicated that M3814-induced activation of ATM and p53 targets can occur effectively in irradiated acute leukemia cells, as in solid tumor cells, reflecting a general mechanism of M3814 intervention in the cellular response to DSB, leading to ATM/p53 potentiation^23^.

We then evaluated the effect of the M3814-enhanced p53 response to IR on cell viability. To this end, the leukemia cell lines were exposed to two doses of radiation (1 Gy and 2 Gy) in the presence of increasing M3814 concentrations and cell growth/viability was assessed after four days (Fig. 1D). While M3814 alone had minimal effect on the cell growth only at high doses, a strong potentiation of the IR effect by M3814 was seen in the p53 wild-type cells but not in p53-deficient cells. Bliss excess analysis^27^ indicated synergism between IR and M3814 in all p53 wild-type but not in the p53-deficient cell lines (Fig. 1D, lower panel).

Next, we tested M3814 for apoptotic activity in combination with IR (2 Gy) in Molm-13 and Molt-4 cells by annexin V staining. Substantially increased apoptotic response was measured 24 h following irradiation in the presence of M3814 in the p53 wild-type leukemia cell lines, Molm-13 and Molt-4, but not in the p53-deficient lines THP-1 and HL-60 (Fig. 1E). M3814 treatment alone did not significantly increase the annexin V signal in any of the four cell lines. IR alone exhibited a moderate induction of apoptosis in a p53-dependent manner. These results demonstrated that M3814 acts as an enhancer of IR-induced p53-dependent apoptosis in leukemia cells.

### M3814 boosts the ATM/p53 response to topoisomerase II inhibitors and sensitizes acute leukemia cells to p53-dependent apoptosis

**Figure 2:**
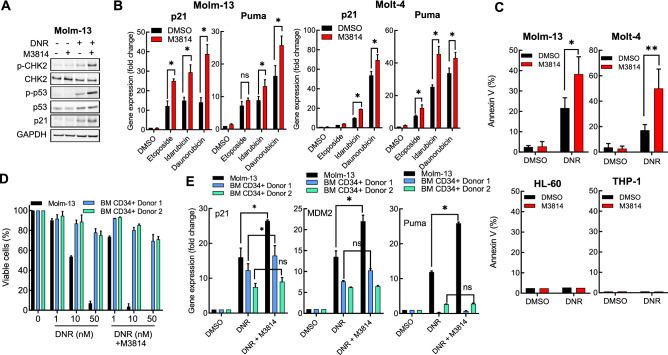
M3814 augments topoisomerase II inhibitor induced p53 apoptotic activity in acute leukemia cells. (**A**) Molm-13 cells were pre-treated with M3814 (300 nM) or vehicle for 45 min before addition of DNR (10 nM) and incubated for additional 24 h before preparation of protein lysates. Protein expression and phosphorylation status were assessed by Western blotting as in Fig. 1A. Panel displays cropped images from repeat blots divided and probed for different targets. Full-sized blots are shown in Supplementary Fig. 2. (**B**) Molm-13 and Molt-4 cells were pre-treated with M3814 (300 nM) or vehicle for 45 min before addition of etoposide (50 nM), idarubicin (1 nM) or daunorubicin (10 nM). RNA was isolated 4 h post-treatment and p53 target gene expression was assessed by qPCR using probes for p21 and Puma. Expression levels were normalized to GAPDH. (**C**) Molm-13, Molt-4, HL-60 and THP-1 cells were pre-treated with M3814 (300 nM) or vehicle for 45 min and exposed to DNR (1 nM). Apoptosis was assessed 24 h post-treatment by flow cytometry as in Fig. 1. D) Molm-13 cells and bone marrow CD34+ cells isolated from two healthy donors were pre-treated with M3814 (300 nM) or vehicle as above for 45 min and DNR (1, 10 or 50 nM) alone or in combination with M3814 (300 nM) was added and the cells were incubated for 4 d. Cell growth/viability was assessed by the CTG assay and expressed as percent of vehicle controls. E) Molm-13 cells and bone marrow CD34+ cells isolated from two independent healthy donors were treated as in D and p53 target gene expression was assessed by qPCR using probes for p21, Mdm2 and Puma. Expression levels were normalized to GAPDH. *p < 0.05, **p < 0.01, ***p < 0.001. NS: p > 0.05.

Topoisomerase II inhibitors such as etoposide and the anthracyclines (doxorubicin, daunorubicin, and idarubicin) have been shown to effectively induce DNA DSB and are widely used in cancer therapy, including acute leukemias^28^. It was previously shown that M3814 synergistically enhances the antitumor effect of topoisomerase II inhibitors in multiple solid tumor cell lines in vitro^22^. We assessed whether DNA-PK inhibition will potentiate the ATM-p53 signaling axis in Molm-13 cells in response to daunorubicin. Daunorubicin treatment alone induced ATM activation resulting in increased levels of its downstream targets, p-CHK2 (Thr^68^) and p-p53 (Ser^15^) 24 h under treatment (Fig. 2A). Co-treatment with DNR and M3814 increased CHK2 and p53 phosphorylation, total p53 and the levels of its direct target p21. To more accurately assess the M3814 effect on p53 downstream signaling, we measured the mRNA levels of the p53 transcriptional target p21 and Puma, key drivers of p53-dependent cell cycle arrest and apoptosis, respectively^29,30^. Two p53 wild-type leukemia lines (Molm-13, Molt-4), were treated with three topoisomerase II inhibitors (daunorubicin, idarubicin and etoposide) for 4 h in the presence or absence of M3814 and mRNA levels of p21 and Puma were determined by qPCR (Fig. 2B). All three inhibitors alone elevated p21 and Puma expression. Addition of the DNA-PK inhibitor further increased the expression of both p53 transcriptional targets.

p53 activation by genotoxic chemotherapeutics or non-genotoxic drugs targeting its negative regulator MDM2 has been established as the main mediator of apoptotic response in acute leukemia^9,28^. Hence, we assessed the apoptosis enhancement effect of the DNA-PK inhibitor on daunorubicin (DNR) in Molm-13, Molt-4, HL-60 and THP-1 cell lines by the annexin V assay after 24 h (Fig. 2C). As expected, M3814 improved the apoptotic response to DNR in the p53 wild-type cells, confirming a p53-driven mechanism.

p53-dependent bone marrow suppression is considered one of the main clinical side effects of topoisomerase II inhibitors. We asked if M3814 addition would further enhance daunorubicin toxicity in human CD34+ cells isolated from the bone marrow of healthy volunteers and propagated in vitro. These cells are believed to represent the stem/progenitor cell compartment of the bone marrow giving rise to all hematopoietic progenitor cells usually affected by DSB-inducing therapies^31^. Proliferating Molm-13 cells, and CD34+ cells from two independent donors, were exposed to increasing concentrations of DNR alone or in combination with M3814 for 4 days and their viability was measured and compared to vehicle controls (Fig. 2D). Under these treatment conditions, bone marrow stem cells were significantly less sensitive to DNR than Molm-13 cells. Co-treatment with M3814 substantially increased DNR cytotoxicity on the AML cells but had a relatively mild effect on the growth/viability of the normal CD34^+^ bone marrow cells. To probe the underlying mechanisms, we measured the changes in the expression levels of the p53 target genes, p21, MDM2 and Puma, in cells exposed to 10 nM DNR in the presence or absence of M3814 (Fig. 2E). As observed previously, DNR treatment induced all three p53 target genes and M3814 enhanced this effect in the AML cells. Bone marrow cells showed induction of p21 and MDM2 genes at a lower level and a slight further increase in the presence of the DNA-PK inhibitor. Interestingly, Puma expression was practically unchanged by DNR or the combination in the cells from both donors, suggesting a potential cause for insensitivity to the DNR + M3814 treatment, since it is a key mediator of p53-dependent apoptosis^19^.

### M3814-enhanced DNR activity is independent of FLT3 mutational status

FMS-like tyrosine kinase 3 (FLT3) is a receptor tyrosine kinase that plays an important role in hematopoietic stem/progenitor cell survival and proliferation. Mutations in FLT3, either internal tandem duplications (ITD) within the juxtamembrane domain or point mutations in the kinase domain, both leading to constitutive kinase activation, are prevalent in AML patients. Approximately one third of AML patients present with FLT3 mutations and they are found predominantly in p53 wild-type AML^32^. Indeed, FLT3-ITD mutations were present in all three wild-type p53 cell lines used in this study (Molm-13, MV4-11, and Molt-4).

**Figure 3:**
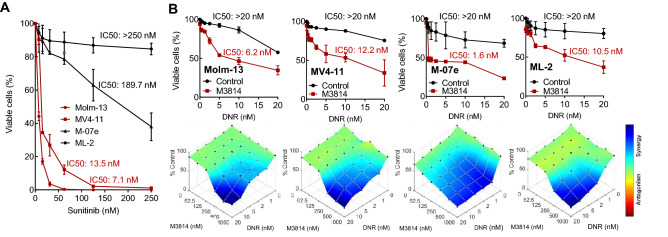
The potentiation of daunorubicin-mediated cell killing by M3814 is independent of FLT3 status. (**A**) Molm-13, MV4-11, ML-2 and M-07e cells were exposed to a dose range of sunitinib and the effect on their growth/viability was assessed by CellTiter-Glo after 3 d. IC50 values were calculated using GraphPad Prism. (**B**) Molm-13, MV4-11, ML-2 and M-07e cells were pre-treated with either DMSO or M3814 (300 nM) for 45 min and then with indicated concentrations of daunorubicin. Cell growth/viability was assessed by CellTiter-Glo on D 4 and IC50 values were calculated as above**.** Shown are daunorubicin dose–response curves (top) and overlays of Bliss synergy on relative viability landscapes (bottom). Bliss synergy was determined using Combenefit software.

To assess if FLT3 status contributes to their sensitivity to the DNR/M3814 combination, we extended the p53 wild-type cancer cell panel with two FLT3 wild-type/p53 wild-type leukemia cell lines, ML-2 and M-07e. As FLT3-ITD cells have been shown to be much more sensitive to sorafenib and sunitinib than FLT3 wild-type AML cells^33^, we first tested the effect of sunitinib on the growth/viability of the four AML cell lines, Molm-13, MV4-11, ML-2, M-07e (Fig. 3A). As expected, FLT3-ITD cell lines, Molm-13 and MV4-11, showed substantially lower IC50 values, hence higher sensitivity to sunitinib, compared to FLT3 wild-type cells. Next, we asked whether FLT3 mutation status confers sensitivity to the DNR/M3814 combination. Cultures from the same four cell lines were exposed to a range of DNR concentrations in the presence or absence of 300 nM M3814 for 4 days. Cell growth/viability was measured and showed comparable potentiation by M3814 regardless of their FLT3 status. These results indicated that the M3814-enhanced effect of DNR is independent of FLT3 mutation status. (Fig. 3B).

### TRAIL-dependent extrinsic apoptosis contributes to enhanced AML cell killing by the DNR/M3814 combination

**Figure 4:**
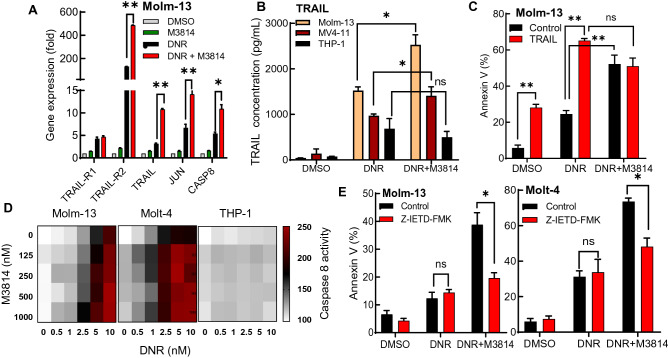
DNR/M3814 combination drives TRAIL-dependent extrinsic apoptosis in leukemia cells. (**A**) Molm-13 cells were pretreated with M3814 (300 nM) or vehicle for 45 min before treatment with 10 nM daunorubicin. RNA was isolated 24-h post-treatment and the expression of TRAIL-R1, TRAIL-R2, TRAIL, JUN, and CASP8 was analyzed by qPCR. Gene expression was normalized to GAPDH. (**B**) Molm-13, MV4-11 and THP-1 cells were treated as described in A. Media from each condition was isolated and assayed by Human TRAIL ELISA. Concentration of TRAIL in medium was determined by a standard curve. (**C**) Molm-13 cells were pre-treated with M3814 (300 nM) or vehicle. Cells were then treated with DMSO or daunorubicin (1 nM) in the presence or absence of TRAIL (50 ng/ml). Apoptosis was assessed 24-h post-treatment by flow cytometry using PE-conjugated annexin V. (**D**) Molm-13, Molt-4 and THP-1 cells were pre-treated with increasing concentrations of M3814 for 45 min. Cells were then treated with DNR in the concentration range 0–10 nM. Caspase-8 activity was measured 6 h post-treatment using the CaspaseGlo-8 Assay and presented as relative luminescence. (**E**) Molm-13 and Molt-4 cells were pre-treated with M3814 (300 nM) or vehicle in the presence or absence of Z-IETD-FAK (10 µM) for 45 min. Cells were then treated with DNR (1 nM). Apoptosis was assessed 24-h post-treatment by flow cytometry using PE-conjugated annexin V. *p < 0.05, **p < 0.01, ***p < 0.001. NS: p > 0.05.

The relatively rapid onset of DNR/M3814 induced cell death in p53 wild-type leukemia cells suggested that extrinsic p53-dependent apoptotic signaling might be involved. To assess its role in leukemia cell death, we measured the expression of several genes known to participate in the induction of extrinsic apoptotic signaling (TRAIL, TRAIL-R1, TRAIL-R2, JUN and CASP8)^34^, in Molm-13 cells exposed to DNR in the presence or absence of M3814 by qPCR (Fig. 4A). Our results revealed that DNA-PK inhibitor can elevate the expression of all genes but TRAIL-R1 and suggested that M3814 potentiates TRAIL signaling induced by DNR. To further elucidate the importance of TRAIL signaling in the mediation of apoptotic response, we quantified the concentration of TRAIL protein released into the media of Molm-13, MV4-11 and THP-1 cells exposed to DNR alone or in combination with M3814 for 24 h (Fig. 4B). DNR induced the release of TRAIL in all three cell lines. Co-treatment with M3814 enhanced TRAIL secretion in the p53 wild-type Molm-13 and MV4-11 cells, but not p53-deficient THP-1.

Next, we examined how treatment of Molm-13 cells with TRAIL alone and in combination with DNR or DNR + M3814 could affect the apoptotic response. TRAIL induced apoptosis in Molm-13 cells at levels similar to DNR (Fig. 4C). Additionally, co-treatment with TRAIL and DNR further increased the apoptotic cell population. Moreover, the TRAIL/DNR combination exerted a similar apoptotic response as the DNR/M3814 treatment. Interestingly, we were not able to enhance the apoptotic output of DNR/M3814 by the addition of TRAIL, suggesting that the TRAIL released from DNR/M3814 treated cells is already saturating the extrinsic apoptotic signal (Fig. 4C).

Subsequently, we evaluated a key component of the downstream death receptor signaling, caspase 8, the extrinsic apoptotic pathway gatekeeper involved in translating apoptotic signals into cell death^35,36^. First, we assessed caspase 8 activity in Molm-13, MV4-11 and THP-1 cells exposed to increasing concentrations of DNR and M3814 in combination after 6 h (Fig. 4D). As depicted in the heatmaps, increasing DNR concentrations elevated caspase-8 activity in Molm-13 and MV4-11 cells. Co-treatment with M3814 further enhanced caspase activity. DNR alone or in combination with M3814 was unable to modulate caspase-8 activity in the p53-deficient THP-1 cells. Then, we asked if blocking the extrinsic pathway with the caspase-8-specific inhibitor, Z-IETD-FM, could rescue the cells from death. The percentage of apoptotic Molm-13 and Molt-4 cells was measured after treatment with DNR alone or in combination with M3814 in the presence or absence of Z-IETD-FM (Fig. 4E). Our results showed that the caspase inhibitor significantly reduced the apoptotic cell fraction induced by the DNR/M3814 treatment in both cell lines, suggesting that caspase-8 activity contributes to the overall apoptotic response. Our result showed that DNA-PK inhibition by M3814 potentiates the p53-dependent apoptosis induced by the DSB-inducing DNR in AML cells and that the extrinsic apoptosis pathway activation via TRAIL is an important component in the overall apoptotic response.

### DNR/M3814 combination promotes AML cell differentiation

**Figure 5:**
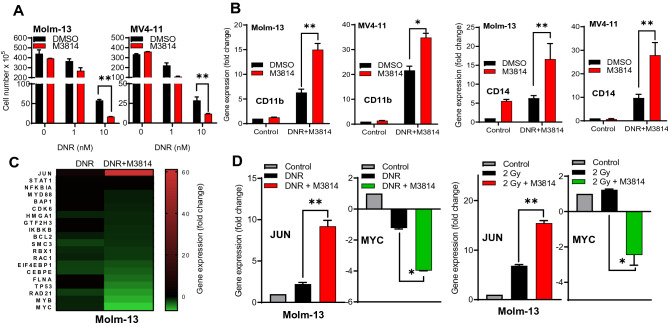
DNR/M3814 combination promotes the myeloid differentiation of AML cells. (**A**) Molm-13 and MV4-11 cells were left untreated or pre-treated with M3814 (300 nM) for 45 min before exposure to DMSO or daunorubicin (1 or 10 nM) and allowed to differentiate for 72 h. Equal number of viable cells were then re-plated in fresh media without inhibitors. Cell number was assessed by Trypan blue exclusion 72-h post-re-plating. (**B**) Molm-13 and MV4-11 cells were pre-treated as in A, then treated with daunorubicin (10 nM) or vehicle. RNA was isolated 24-h post-treatment. and the expression of the myeloid markers CD11b and CD14 was quantified by RT-qPCR. mRNA expression was normalized to GAPDH. (**C**) Molm-13, cells were pre-treated as in A, then treated with daunorubicin (10 nM) or vehicle. Gene expression was assessed using the nCounter Pan-Cancer Pathway Panel after 24 h. The changes in expression of a subset of genes previously associated with myeloid differentiation was displayed on a heatmap as a fold change over vehicle-treated Molm-13 cells. (**D**) Molm-13 cells were pre-treated as in A. Cells were then treated with DMSO, daunorubicin (10 nM) or IR (2 Gy). RNA was isolated 24-h post-treatment and RT-qPCR was used to quantify the expression of JUN and MYC. mRNA expression was normalized to GAPDH. *p < 0.05, **p < 0.01, ***p < 0.001. NS: p > 0.05.

It has been hypothesized that AML development is triggered by a block of myeloid differentiation and reversing it may allow cancer cells to proceed to their fully differentiated state^37,38^. We assessed whether DNR/M3814 induced p53 overactivation could affect the differentiation status of AML cells. Molm-13 and MV4-11 cells were exposed to M3814 (300 nM) in combination with 1 nM or 10 nM DNR for 3 days, re-plated in media without inhibitors and allowed to grow for another 3 days. At the end of treatment, viable cell number was determined by trypan blue exclusion. Cells exposed to M3814 alone had growth rate comparable to the vehicle control (Fig. 5A). DNR alone inhibited cell growth/viability whereas co-treatment with M3814 further substantially reduced the fraction of viable cells. Then, we assessed the ability of DNR/M3814 to promote myeloid differentiation of AML cells. We first examined mRNA expression of two key myeloid differentiation markers, CD11b and CD14, in Molm-13 and MV4-11 cells (Fig. 5B). While DNR alone increased the expression of both markers in both cell lines, co-treatment with M3814 further enhanced their expression, indicating that DNA-PK inhibitor acts as a booster of DNR induced AML differentiation. M3814 alone showed no effect on CD11b expression in both cell lines but increased CD14 expression in Molm-13 cells.

Looking for underlying mechanisms, we examined changes in gene expression of protein factors previously associated with hematopoietic cell differentiation in Molm-13 cells exposed to M3814/DNR or DNR alone using the NanoString nCounter Pan-Cancer Pathway Panel. Highlighted in Fig. 5C, are M3814/DNR induced changes in expression in a subset of genes reported to play a regulatory role in AML. JUN, an important promoter of myeloid differentiation^38^, was found to be the most highly upregulated gene following 24-h treatment with the DNR/M3814 combination. DNR alone augmented JUN expression to a much lesser extent (Fig. 5C) while M3814 alone had no effect on JUN expression (data not shown). The expression levels of modulators of the JNK/cJun pathway such as RBX1 and RAC1^39^ were increased nearly 20-fold by the DNR/M3814 combination. IKBKB and MYB88, key signaling mediators of myeloid differentiation (^40^), were also upregulated by the DNR/M3814 combination. However, no differences in gene expression between DNR alone and DNR/M3814 for other mediators of differentiation such as STAT1, NFKB1A were found. MYC and MYB were the most down-regulated genes upon DNR/M3814 treatment. Interestingly, MYC and MYB have been implicated in the differentiation block and proliferation of hematopoietic cells^40,41^. The modulation of MYC and JUN expression in Molm-13 cells was confirmed by qPCR (Fig. 5D). Addition of M3814 increased the expression of JUN and decrease the expression of MYC in response to DNR treatment. These results support a role of the DNA-PK inhibitor in promoting higher level of AML cell differentiation in response to DSB-inducing DNR.

### M3814 enhances the SoC efficacy in AML cells and a patient-derived mouse model

**Figure 6:**
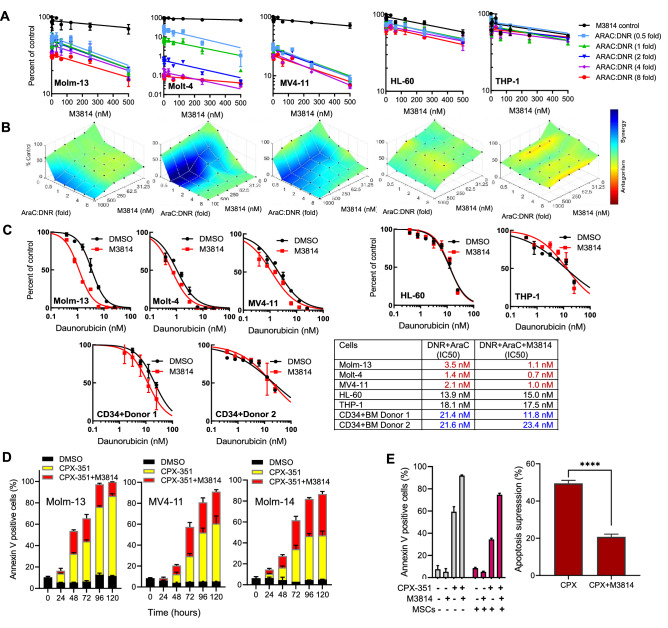
M3814 enhances the effect of DNR/AraC combination therapy in acute leukemia cells. (**A**) Molm-13, Molt-4, MV4-1, THP-1 and HL-60 cells were pre-treated with the indicated concentrations of M3814 or vehicle for 45 min before the addition of 1 nM DNR and increasing concentrations of Ara-C at AraC:DNR ratios: 0.5, 1, 2, 4 and 8. Cell viability was assessed by the CellTiter-Glo assay on D 4. (**B**) Combination activity in the above treatment was assessed by the Bliss synergy method using Combenefit software. (**C**) Molm-13, Molt-4, MV4-11, THP-1, HL-60 and BM CD34 + cells isolated from two independent healthy donors were pre-treated with M3814 (300 nM) for 45 min followed by addition of increasing DNR concentrations in combination with a fixed dose of Ara-C (50 nM). Cell viability was assessed on day 3 by the CellTiter-Glo assay. IC50 values were calculated using GraphPad Prism. Representative data from three independent experiments is shown. (**D**) MOLM-13, MV4-11 and MOLM-14 cells were treated with CPX-351 (62.5 nM) with or without M3814 (500 nM) for indicated treatment times. Percentages of annexin V positive cells were determined by flow-cytometry and plotted as stacked bars. (**E**) Left: MOLM-13 cells were cultured with or without MSC and treated with CPX-351 (62.5 nM) with or without M3814 (500 nM). 25,000 MSC were seeded one day before starting co-culture. Percentages of annexin V positive cells in MOLM-13 cells were determined by flow-cytometry. Right: Inhibition of apoptosis in MOLM-13 cells co-cultured with MSC and treated with CPX-351 with or without M3814. **** p < 0.0001.

In the treatment of AML, DNR is administered in combination with AraC^42^. Therefore, we asked whether M3814 could also potentiate the response of leukemia cells to the DNR/AraC combination. To this end, the panel of five acute leukemia lines with different p53 status (MOLM-13, MV4-11, Molt-4, HL-60, THP-1) were exposed to varying concentrations of M3814 at different AraC: DNR ratios (1:2, 1:1, 2:1, 4:1, 8:1) and their growth/viability was determined by the CTG assay (Fig. 6A). As expected, M3814 enhanced DNR effects on cell growth/viability only in p53 wild-type cells (Molm-14, MV4-11, and Molt-4) but not p53-deficient cells (HL-60, THP-1). The calculated Bliss scores indicated a synergistic relationship between DNR and M3814 in a p53-dependent manner (Fig. 6B). However, the effects of M3814 on DNR-mediated cytotoxicity were independent of AraC, as increasing AraC concentrations showed no significant effect on cell viability. This is in line with our previous findings showing no synergy between M3814 and AraC in screening experiments using multiple cancer cell lines^22^.

Next, we assessed the potentiation of antitumor activity of DNR/AraC by M3814 and its effect on the viability of normal bone marrow stem (CD34 +) cells cultivated in vitro. Molm-13, Molt-4, MV4-11, THP-1, HL-60, and BM CD34 + cells isolated from two independent healthy donors were exposed to 50 nM AraC in combination with a range of DNR concentrations in the presence or absence of 300 nM M3814 and cell growth/viability was assessed after 72 h (Fig. 6C). M3814 potentiated the effects of DNR/AraC in p53 wild-type, but not p53-deficient cells. More importantly, the potentiation effect of M3814 was more pronounced in p53 wild-type AML cells compared with normal BM CD34+ cells.

Recently, CPX-351 (Vyxeos), a liposomal formulation of fixed AraC/DNR ratio (5:1) was approved by FDA for treatment of secondary AML, based on its superior clinical activity compared to conventional AraC/DNR^37,43^. In combination with CPX-351, M3814 enhanced the apoptotic response in AML cells with wild-type p53 (Fig. 6D). To assess the impact of M3814 on the protective effect of bone marrow microenvironment on AML cells, we co-cultured MOLM-13 cells with mesenchymal stromal cells (MSC) derived from healthy bone marrow donors and exposed them to M3814 and CPX-351 (Fig. 6E). The combination treatment enhanced the apoptotic response of AML cells even under co-culture conditions. The MSC co-culture inhibited drug-specific apoptosis by 50% in CPX-351 monotherapy but only by 20% in the combination treatment, suggesting that the synergistic effect of M3814 in combination with CPX-351 is only marginally diminished by the protective effect of MSCs on AML cells.

**Figure 7:**
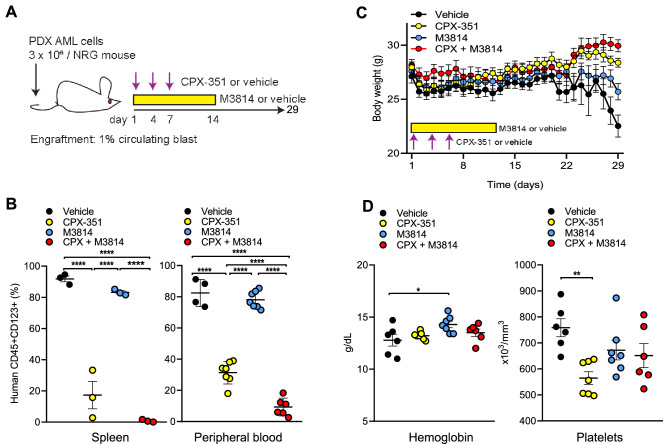
Combination treatment with CPX-351 and M3814 is tolerable and induces profound cytoreduction compared to CPX-351 alone in vivo. (**A**) In vivo treatment scheme. Mice were injected with 3 × 10^6^ PDX AML cells and after engraftment (defined as > 1% circulating human CD45 and CD123 double positive cells) were randomly assigned to four groups (N = 10 each) and treated with vehicle, M3814 (oral gavages, 25 mg/kg twice a day for 14 days), CPX-351 (intravenous injections, 5 U/kg on days 1, 4 and 7) and M3814 + CPX-351. M3814 was formulated weekly in 0.5% Methocel A4M (Sigma 94,378), 0.25% Tween 20 and 300 mM sodium citrate. The leukemia burden was monitored as percentages of human CD45 and CD123 double positive cells in peripheral blood collected by retroorbital bleeding. After treatment for the indicated time, three mice from each group were randomly selected and euthanized by CO_2_ asphyxiation and cervical dislocation on day 21, when mice in the vehicle-treated group became moribund. Percentages of human CD45 and CD123 double positive cells were measured by flow-cytometry. (**B**) Left: Percentages of human CD45 + CD123 + blasts in spleens for each treatment group on day 21. Right: Percentages of human CD45 + /CD123 + circulating blasts for each treatment group on day 28. Ordinary one-way ANOVA analysis was used for multiple comparisons. (**C**) Body weight during treatment courses for each group. (**D**) Hemoglobin and platelets levels for each treatment group on day 28.

Next, we investigated the efficacy of the CPX-351/M3814 combination in vivo. NRG mice (retaining an intact *PRKDC* gene) were injected with AML cells obtained from a patient whose AML relapsed after four prior treatments and stem cell transplantation. After engraftment, mice were treated with either vehicle, M3814, CPX-351, or CPX-351 + M3814 (Fig. 7A). The combination treatment markedly reduced human CD45^+^CD123^+^ cells in animal spleens and circulating CD45^+^CD123^+^ blasts compared with vehicle, M3814 and CPX-351 monotherapies (Fig. 7B). During the 29-day observation period, the combined CPX-351/M3814 treatment showed no significant body weight changes (Fig. 7C) or reduction in hemoglobin and platelets levels (Fig. 7D), suggesting that addition of DNA-PK inhibitor to the optimized chemotherapy formulation CPX-351 provides a superior anti-leukemia effect while sparing normal hematopoietic cells.

## Discussion

*TP53* mutations are rare in AML and infrequently considered drivers of malignancy. Indeed, less than 10% of AML patients present with *TP53* mutations^44,45^. *TP53* mutations in the general AML population are commonly acquired following treatment and are associated with insensitivity to classical therapies^46^. Altogether, clinical evidence suggests an important role of p53 in mediating the therapeutic responses in AML. Induction therapy with AraC and anthracyclines (daunorubicin or idarubicin) remains the SoC for younger patients with AML. However, many patients treated with SoC relapse or experience several drug-related toxicities^6^. Anthracyclines have been reported to induce p53-dependent apoptosis in normal hematopoietic cells. Thus, both the efficacy and toxicity associated with this class of drugs is often driven by the engagement of the p53 pathway as part of the DNA damage response both in cancer and normal cells^47^.

The p53 tumor suppressor integrates multiple cellular signals and stresses to either promote cell cycle arrest, DNA repair or apoptosis^18,48^. Unlike in many solid tumors, which commonly evade apoptosis through mutations in p53 or disabling p53 dependent apoptotic signaling in p53 wild-type tumors^25^, most treatment naïve AML patients present with wild-type p53 tumors^46,47^. These tumors are sensitive to p53-dependent chemotherapy^9^, suggesting that p53 apoptotic signaling is still functional and they are capable of undergoing p53-dependent apoptosis. Indeed, treatment of AML with MDM2 antagonists, which stabilize and activate p53, has been shown to promote p53-dependent apoptotic AML cell death^9,10^.

Here, we explore a potential novel therapeutic option for enhancing leukemia cytotoxicity of DSB-induced p53 activation in AML. We show that the inhibition of DNA-PK with the selective inhibitor, M3814, potentiates the ATM/p53 response to DNA DSB induced by IR or topoisomerase II inhibitors (e.g., etoposide, daunorubicin, and idarubicin) in acute leukemia cells, leading to increased p53-dependent apoptosis. This potentiation has been demonstrated with DNR alone or with DNR/AraC combination supporting its potential use for improving the SoC treatment in AML. Our results were obtained with small panels of p53 functional and dysfunctional cell lines rather than isogenic p53-wt/p53-null pairs and one could not exclude the possibility of p53-independent events. However, the clear differences in response along the p53 status line (Figs. 1, 2, and 5) together with the previously established p53-dependent mechanism in solid tumor cell lines exposed to IR and M3814^23^ strongly support the hypothesis that the same fundamental mechanism is responsible in acute leukemia cells. We recently reported that ATM/p53 overactivation is behind the synergy between M3814 and another DSB-inducing agent, calicheamicin, in the wild-type p53 AML cell line Molm-13 in vitro and in vivo, suggesting a new combination approach to improving AML treatment with Mylotarg^26^.

DNA-PK inhibitors have been developed to block DSB repair and have proven very effective in enhancing the antitumor efficacy of IR and DSB-inducing chemotherapy, primarily by inducing mitotic catastrophe^15,16^. The overactivation of the ATM/p53 axis by M3814 in combination with DNR offers an additional lever for enhancing cancer cells death in acute leukemia. The overheated p53 response to DSBs leads to reinforced cell cycle arrest and premature senescence in p53 wild-type solid tumor cells, protecting them from apoptotic death^23^. However, it effectively boosts p53-dependent apoptosis induced by SoC in AML, the main mechanism for clearance of the predominantly p53 wild-type leukemia cells. Therefore, DNA-PK inhibitor is well suited for combinations with DSB-inducing agents in acute leukemia.

DNA damage has previously been shown to promote the differentiation of AML cells^49^. Additionally, modulation of p53 expression and activity can also mediate AML differentiation^50,51^. We showed that DNR treatment increases the expression of myeloid differentiation markers compared to untreated control cells. M3814 further augmented the expression of the myeloid markers, CD11a and CD14, suggesting that DNA-PK inhibitor could provide two potential benefits in AML therapy: enhance DNR-induced apoptosis as well as myeloid differentiation. Myeloid differentiation also plays an important role in mediating AML relapse. Leukemia stem cells (LSC) have been reported as a key contributor to disease relapse and resistance to SoC therapy in AML. LSCs are characterized by multipotency, ability to self-renew and high proliferative capacity. Thus, driving LSC to a more differentiated state via the DNR/M3814 combination may support more favorable patient outcomes. Further studies on the specific molecular events behind the differentiation enhancing effect of M3814 are warranted and could shed more light on the underlying mechanisms.

The use of chemotherapeutics such as DNR in AML is often detrimental to the normal marrow tissue causing injury to the hematopoietic microenvironment and bone marrow suppression^52^. MDM2 antagonists have been introduced as an alternative to classical DNA-damaging chemotherapies to spare normal tissue from the genotoxic insult^8,9,53,54^. However, clinical studies revealed that the approach has also been plagued with p53-dependent bone marrow toxicity^8,55^. While M3814-enhanced p53 response to DSBs was shown to potentiate AML cell killing, it appears less toxic to normal bone marrow CD34^+^ stem cells in vitro* and* in vivo. The resistance of normal bone marrow cells to M3814-enhanced p53 activation might be due to ineffective induction of Puma, a key mediator of p53-dependent apoptosis^9,20^. However, one could argue that CD34+ bone marrow cells propagated in vitro have limited value as predictors of treatment outcome as they grow slower compared to AML cell lines. Nevertheless, the M3814-based cytotoxic combination appeared to be well tolerated in vivo.

Our studies highlight a novel combination strategy for treatment of AML with increased efficacy and possibly improved safety. We demonstrate that DNA-PK inhibition significantly improves the apoptotic response of AML cells to treatment with DNR/AraC while being less detrimental on normal BM cells in vitro. Also, the drug combination could counter the differentiation block in AML cells and promote their myeloid differentiation, possibly minimizing the fraction of undifferentiated leukemic stem cells often present in relapsing patients treated with the SoC. More effective activation of the apoptotic pathway in AML could also reduce chemoresistance.

CPX-351, a liposome-encapsulated drug combining DNR and AraC with a fixed ratio (5:1), has improved pharmacokinetic activity in blood and bone marrow compared to the free drugs^56^, and recently shown to yield higher complete remission rates and prolonged survival in patients with secondary AML^43^. However, more than half of the patients who received CPX-351 relapse within 12 months, with a 31.1% survival rate at 2 years, warranting the search for improved treatment strategies. In the current study, the combination of M3814 with CPX-351 exerted superior cytoreduction compared to CPX-351 monotherapy in vitro and in vivo, with no additional toxicities in mice. This might be helped by the improved uptake of CPX-351 in marrow leukemia cells^57^ allowing for enhanced and more selective activity of M3814 in combination with CPX-351 in AML versus normal bone marrow cells. The PDX AML model in the current study was developed from a patient who had four prior therapies including SCT, supporting a clear rationale for clinical investigation of this combination treatment, especially in high-risk or relapsed AML patients. Currently, CPX-351 is under investigation in non-treatment related AML, suggesting potentially broader impact of the proposed combination therapy.

## Methods

### Reagents

The DNA-PK inhibitor, M3814, was synthesized at Merck KGaA, Darmstadt, Germany as described. CPX-351 (Vyxeos®) was provided by Jazz Pharmaceuticals as part of a grant from CTEP. Daunorubicin, etoposide, idarubicin, sunitinib, and Z-IETD-FMK were purchased from Selleckchem (Houston, TX). Arabinoside (Cytarabine, AraC) and TRAIL were purchased from MilliporeSigma (Burlington, MA). All inhibitors were dissolved in DMSO to prepare a 10 mM stock and stored at − 20 °C. Aliquots were diluted directly in tissue culture media containing 10% FCS to a desired concentration. The final concentration of DMSO in media did not exceed 0.11% (vol/vol) which has not shown detectable effect on cell morphology, viability and differentiation potential.

### Cell lines and tissue culture

The following cell lines were used in this study: p53 wild-type/FLT3ITD: MOLM-13 (adult acute myeloid leukemia), MV4-11 (adult acute myeloid leukemia, MOLT-4 (acute lymphoblastic leukemia). p53 wild-type/FLT3 wild-type: ML-2 (adult acute myeloid leukemia), M-07e (adult acute myeloid leukemia). p53-null/mutant: HL-60 (adult acute myeloid leukemia), THP-1 (adult acute myeloid leukemia). They were purchased from ATCC (Manassas, VA) or DSMZ (Braunschweig, Germany) and maintained in RPMI 1640 media supplemented with 20% heat-inactivated fetal bovine serum (FBS). M-07e cells were cultured in RPMI 1640 supplemented with heat-inactivated fetal bovine serum and GM-CSF (10 ng/ml). All cell lines were maintained at low passage and confluence. Short tandem repeats (STRs) were analyzed to confirm cell line identity and mycoplasma infection was excluded by a PCR-based testing. Human bone marrow (BM) CD34 + cells obtained from healthy donors were purchased from StemCell Technologies, Inc. (Vancouver, BC, Canada) and maintained in StemPro-34-SFM media from GIBCO (Waltham, MA). They were maintained for up to 7 days in liquid culture media. Healthy bone marrow donor-derived MSC were collected under MD Anderson Cancer Center Institutional Review Board approval (LAB02-0395). Human BM-derived MSCs were isolated as described previously^58^.

### MSD assay and Western blot analysis

MSD assays for phospho-ATM (S1981) and total ATM were performed as previously described^17^. Capture antibodies were ab208775 (Abcam Biotechnology, Cambridge, MA) for phospho-ATM (S1981) and sc-135663 (Santa Cruz Biotechnology, Dallas, TX) for total ATM. First detection antibodies were sc-135663 (Santa Cruz Biotechnology, Dallas, TX) for phospho-ATM (S1981) and ab199726 (Abcam Biotechnology, Cambridge, MA) for total ATM. Second detection antibodies were R32AC, MSD SULFO-TAG labeled anti-mouse (MSD, Gaithersburg, MD) for phospho-ATM (S1981) and R32AB, MSD SULFO-TAG labeled anti-rabbit (MSD, Gaithersburg, MD) for total ATM. Cells were lysed in RIPA buffer (Thermo Fisher Scientific, Waltham, MA) supplemented with protease and phosphatase inhibitors (Roche Diagnostics, Indianapolis, IN). Lysates were resolved using NuPAGE 4–12% Bis–Tris, or 3–8% Tris–Acetate gels (ThermoFisher Scientific) and transferred to Nitrocellulose membranes with an iBlot 2 Gel Transfer Device (ThermoFisher Scientific). Membranes were divided and probed for different target proteins and imaged with a LI-COR Odyssey CLx imaging system in accordance with the LI-COR Near-Infrared (NIR) Western Blot Detection Protocol (LI-COR, Lincoln, NE). Primary antibodies were as follows: KAP1 (#ab22553, Abcam Biotechnology, Cambridge, MA); p-KAP1 (S824), (#ab133440, Abcam Biotechnology); p-CHK2 (T68) (#2197, Cell Signaling Technology, Danvers, MA); CHK2 (#3440 and #6334, Cell Signaling Technology); p-p53 (S15) (#9284, Cell Signaling Technology); p53 (#48818, Cell Signaling Technology); Puma (#12450, Cell Signaling Technology); Cleaved Caspase 3 (#9664, Cell Signaling Technology) and GAPDH (#sc47724, Santa Cruz Biotechnology, Dallas, TX).

### mRNA quantification

RNA was isolated using Trizol (ThermoFisher Scientific) extraction. RNA purity and concentration were determined using Nanodrop (ThermoFisher Scientific). For RTqPCR experiments, reverse transcription was completed using the Superscript IV Vilo Master Mix (ThermoFisher Scientific) as described by manufacturer. Quantitative PCR was performed using the Taqman Fast Advanced Master Mix and indicated Taqman probes (Applied Biosystems, Foster City, CA) and ran on a 7500 Fast Dx Real-Time PCR Instrument (Applied Biosystems). Target gene expression was normalized to GAPDH. For high-throughput mRNA quantification, 50 ng of isolated RNA samples were assessed using the nCounter PanCancer Pathway Panel (NanoString, Seattle, WA) according to manufacturer’s instruction.

### Growth/viability and apoptosis assays

For growth/viability testing, 1000–2500 cells were plated overnight in 96-well plates. The next day, cells were pre-treated with DMSO (control) or increasing doses of M3814 for 45 min, then either irradiated with indicated dose and/or treated with a dose range of daunorubicin for 3–4 days. Viability was assessed using the CellTiter Glo 2.0 assay (Promega, Madison, WI) according to manufacturer’s instruction. Luminescence was detected using the EnVision plate reader (Perkin Elmer, Waltham, MA). Concentration–response curves and IC50 values were generated by graphing relative viability and curve fitting using GraphPad Prism (v8.0.1). For apoptosis assays, cells were plated overnight in 6-well plates. Cells were then pre-treated with M3814 (300 nM) for 45 min then either irradiated at 1 Gy or treated with daunorubicin (1 nM). Cells undergoing apoptosis were detected at 24 h by flow cytometry using the Annexin V-PE staining kit (BD Biosciences, Billerica, MA). Bliss scores were determined using data acquired by the CellTiter Glo 2.0 assays described above and calculated using Combenefit software^27^. For caspase 8 activity, 5000 cells were plated in 96-well plates and incubated overnight. Cells were then treated with increasing concentrations of M3814 for 45 min and then treated with increasing concentrations of daunorubicin. The CaspaseGlo 8 Assay System (Promega) was used to quantify caspase 8 activity at 6 h. Luminescence was measured with the EnVision plate reader.

### TRAIL ELISA assay

Cells were pre-treated with M3814 (300 nM) for 45 min followed by incubation in daunorubicin (10 nM) or vehicle and cell media was collected 24 h later. Secreted TRAIL was quantified using the Human TRAIL ELISA kit (ThermoFisher Scientific) as described in manufacturer’s instructions.

### AML differentiation assays

Cells were pre-treated with M3814 (300 nM) for 45 min before addition of 10 nM daunorubicin and incubated for 24 h. RNA was isolated by Trizol extraction and the expression of myeloid differentiation markers (JUN, MYC, CD11b and CD14) was assessed using RT-qPCR as described above.

### Animal model

The animal study was performed in accordance with the protocol approved by MD Anderson Cancer Center Institutional Animal Care and Use Committee (00001303-RN01). Six to eight-week-old male NOD.Cg-Rag1tm1Mom Il2rgtm1Wjl/SzJ (NRG) mice (Jackson Laboratory), with intact *PRKDC* gene received 450 cGy whole-body radiation followed by intravenous injections with 3 × 10^6^ cells/mouse derived from a PDX AML model established at MDACC (UPIN: 3912018) with wild-type *TP53*. The study was carried out in compliance with the ARRIVE guidelines. Details about the PDX model could be found in Supplementary Information.

### Statistical analyses

Calculation of IC50 values and statistical tests were performed with GraphPad PRISM version 8.0 (GraphPad Software Inc.) as previously described. The data were analyzed with Student *t* tests. *P* values ≤ 0.05 were considered statistically significant. All assays were conducted independently at least three times, unless indicated otherwise, and representative data is shown as mean ± SD. Significance values are *p < 0.05, **p < 0.01, and ***p < 0.001. NS stands for non-significant (p > 0.05).

## Supplementary Information

Supplementary Information.

## Data Availability

The datasets generated during and/or analyzed during the current study are available from the corresponding author on reasonable request.
