# Supplementary material for: DNA-PK inhibitor peposertib enhances p53-dependent cytotoxicity of DNA double-strand break inducing therapy in acute leukemia
Source: Sci Rep. 2021 Jun 9;11:12148. doi: 10.1038/s41598-021-90500-3 (PMC8190296; doi:10.1038/s41598-021-90500-3)
Supplement: Supplementary file 1 — Supplementary Information. [file 41598_2021_90500_MOESM1_ESM.pdf]

DNA-PK inhibitor peposertib enhances p53-dependent cytotoxicity of DNA double-strand break inducing therapy in acute leukemia

Eric Haines[1*], Yuki Nishida[2*], Michael I. Carr[1], Rafael Heinz Montoya[2], Lauren B. Ostermann[2], Weiguo Zhang[2], Frank T. Zenke[3], Andree Blaukat[3], Michael Andreeff[2†*], and Lyubomir T. Vassilev[1†*]

[1]Translational Innovation Platform Oncology and Immuno-oncology, EMD Serono Research & Development Institute, Inc., Billerica, MA, USA, an affiliate of Merck KGaA, Darmstadt, Germany

[2]Section of Molecular Hematology and Therapy, Department of Leukemia, The University of Texas MD Anderson Cancer Center, Houston Texas, USA,

[3]Translational Innovation Platform Oncology and Immuno-oncology, Merck KGaA, Darmstadt, Germany.

*These authors contributed equally to the work

[†]Corresponding authors:

Lyubomir T. Vassilev, EMD Serono Research & Development Institute, Inc., 45A Middlesex Turnpike, Billerica, MA 01821, USA, e-mail: lubo.vassilev@emdserono.com; and

Michael Andreeff, Section of Molecular Hematology and Therapy, Department of Leukemia, The University of Texas MD Anderson Cancer Center, 1515 Holcombe Blvd., unit 448, Houston, TX 77030, e-mail: mandreef@mdanderson.org.

Supplementary Information

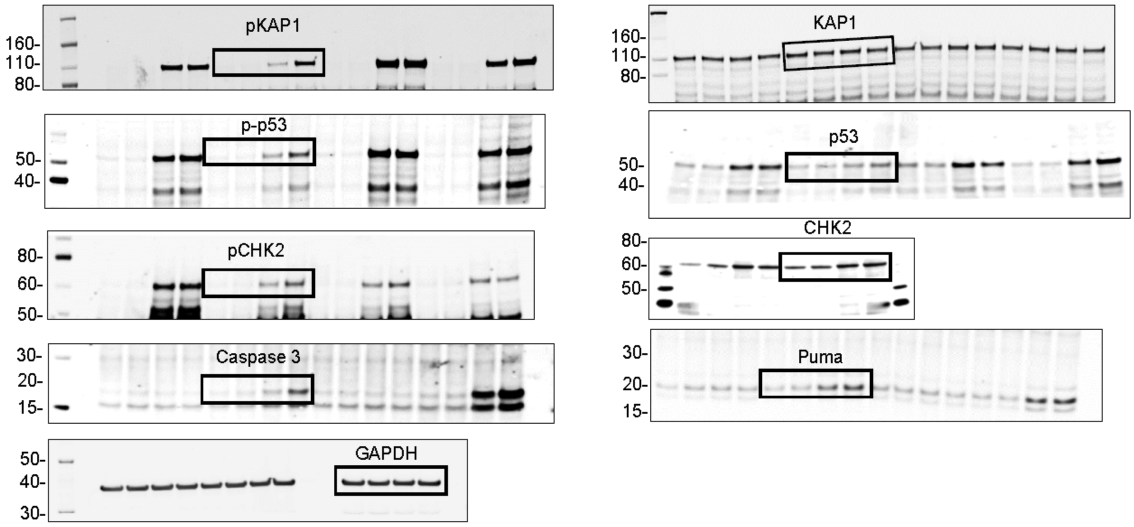

Figure S1. Images of full-size immunoblots corresponding to data shown in Fig. 1A, with cropped areas defined. Repeat blots were divided and probed for different targets. Numbers represent protein molecular weight standards in kDa. Black lines indicate edges of acquired images of individual membrane segments using a LI-COR Odyssey CLx imaging system.

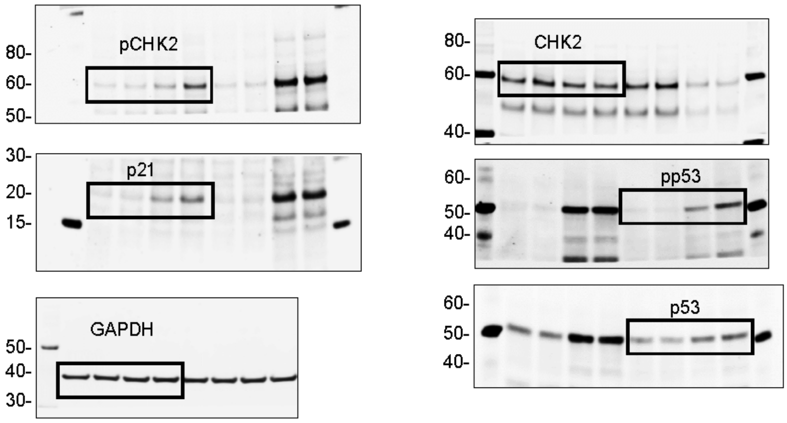

Figure S2. Images of full-size immunoblots corresponding to data shown in Fig. 2A, with cropped areas defined. Repeat blots were divided and probed for different targets. Numbers represent protein molecular weight standards in kDa. Black lines indicate edges of acquired images of individual membrane segments using a LI-COR Odyssey CLx imaging system.

Clinical information of PDX AML model used in the study

General information: 48 y.o. Female

Disease status: relapsed AML

Bone marrow blasts: 82%

Cytogenetics: 46,XX (only obtained at the time of diagnosis)

Gene mutations: FLT3-ITD (allelic ratio 0.016), DNMT3A (p.R882S), IDH1 (p.R132H), KIT (p.M541L) and NPM1 (p.W288fs)

Prior treatments before sample collection: 3 + 7 (CR, 5 years), FLAG-IDA, CIA (CR for 5 months followed by stem cell transplantation) and SGI110.
